# Mitigating the Health Effects of Aqueous Cr(VI) with Iron-Modified Biochar

**DOI:** 10.3390/ijerph19031481

**Published:** 2022-01-28

**Authors:** Zhihong Zheng, Xiaohan Duan

**Affiliations:** 1 School of Water Conservancy, North China University of Water Resources and Electric Power, Zhengzhou 450046, China; zzh@ncwu.edu.cn; 2 Henan Vocational College of Water Conservancy and Environment, Zhengzhou 450008, China

**Keywords:** adsorption, environmental health, chromium, ferric chloride, traditional Chinese medicine residual

## Abstract

A large amount of chromium (Cr) has entered the natural environment from the wastewater and waste residues, and the hexavalent (Cr(VI)) is highly poisonous, threatening the ecological environment and human health directly. In this study, iron-modified biochar was prepared using honeysuckle residue as raw material and the ferric chloride impregnation method. Batch Cr(VI) adsorption experiments were carried out using the modified honeysuckle-derived biochar (MHDB) as an adsorbent. The results indicate that a pH of 2 was best for the adsorption removal of Cr(VI) in the initial pH range of 2–10. The adsorption kinetic data fitted the pseudo-second-order model best out of the two models, and the Langmuir model was better than the Freundlich model to describe the adsorption process. Thermodynamic analysis indicated that the adsorption process of Cr(VI) on MHDB had an endothermic and spontaneous nature, and the increasing temperature was conducive to the adsorption. The main mechanisms of Cr(VI) adsorption might be the physical adsorption (electrostatic interactions) and chemical adsorption (ion exchange, the reduction of Cr(VI) to Cr(III)). The efficient adsorption of Cr(VI) makes MHDB a potential material for Cr(VI)-containing wastewater treatment. This study provides a feasible adsorption material for mitigating the environmental hazards of chromium, which has a certain reference value for protecting environmental health.

## 1. Introduction

Heavy metal pollutants have raised worldwide concerns because of their toxicity and persistence, causing an indefinite threat to the sustainable development of environmental health and human safety [1,2,3,4]. Among these heavy metals, chromium (Cr) is a commonly known pollutant derived from various industries, such as steel manufacturing, dye manufacturing, leather tanning, oil color, and electroplating [5,6,7,8,9,10]. Cr usually exists in the forms of trivalent (Cr(III)) and hexavalent (Cr(VI)) [11]. Cr(III) and Cr(VI) can be converted to each other under certain conditions [12]. Cr(III) is a micronutrient with low solubility and mobility that can be easily removed from water in the form of stable and insoluble precipitates [13]. In contrast, Cr(VI) is several hundred times more toxic than Cr(III) due to its high toxicity, higher migration, and non-biodegradability [14,15]. Cr(VI) has much higher mobility in water and soil and can be transformed into various reactive and toxic intermediates, eventually endangering human health because of the accumulation characteristics [16,17]. Cr(VI) can cause cell mutations and lead to skin injury, intestinal diseases, kidney diseases, cancer, and other diseases [18,19,20,21]. Consequently, Cr(VI) is a significant threat to the quality of water, soil environments, and human health and has been considered a top-priority pollutant to be addressed in water remediation [22]. To minimize the environmental risk caused by Cr(VI) and ensure human health, the maximum levels of chromium allowed in drinking water must not outnumber 0.05 ppm according to the World Health Organization (WHO) [23]. Therefore, it is of great importance to remove Cr(VI) from water safely and efficiently.

Various techniques, including chemical precipitation, ion exchange, ultrafiltration, membrane separation, adsorption, and so on, have been developed to remove Cr(VI) from water [24,25,26]. Among the techniques, adsorption is a promising method for Cr(VI) removal due to its low cost, simple operation, and high efficiency [27,28]. Biochar, considered one of the most promising adsorbents because of its low cost and excellent adsorption effect, has been used for the removal of chromium-containing wastewater successfully to protect environmental health [29,30]. Biochar (BC) is obtained by the pyrolysis of biomass that is rich and diverse. Biomass made up of bagasse, corn stalk, peanut shell, oak wood, sludge, and rice straw has been used for the preparation of biochar successfully [31,32,33,34,35].

With the development of traditional Chinese medicine in recent years, plenty of traditional Chinese medicine residuals (TCMRs) have been created. Up to 70 Mt of TCMRs are produced per year in China. Owing to the limited availability of effective recycling methods, TCMRs are mainly disposed of as solid waste in landfill [36]. Thus, using TCMRs as a raw material with which to prepare biochar is a promising method for its reclamation. According to the statistical data, honeysuckle is a widely used traditional Chinese medicine, which showed the best adsorption effect of Cr(VI) among ten kinds of TCMRs, including astragalus membranaceus, radix Isatidis, thorowax, and others in our pre-experiment.

In order to enhance the adsorption performance of biochar, researchers often use surface modification methods to improve the pore structure and the surface functional groups, such as chemical oxidation, chemical reduction, and metal impregnation [37,38]. Ferric chloride (FeCl_3_) is a common chemical reagent that can provide hydrogen ions in aqueous solutions. Previous studies have indicated that modification using FeCl_3_ could increase the mesopores of carbon materials and that Fe oxides have great selectivity and affinity for Cr(VI) [39]. Hence, FeCl_3_ was chosen as a modifier to promote the Cr(VI) adsorption capacity in this study and, thus, to better reduce the harm of Cr(VI) in the environment.

Therefore, in order to prepare an effective biochar material to solve the problem of Cr(VI) in the environment, honeysuckle residue was used as a raw material to prepare honeysuckle-derived biochar (HDB), and the ferric chloride impregnation method was used to obtain modified honeysuckle-derived biochar (MHDB) to improve the adsorption capacity. The influencing factors of Cr(VI) adsorption were investigated to explore the adsorption mechanism. The study could be a valuable reference for the resource recovery of TCMRs and provide a resolvent for chromium-containing wastewater while alleviating the environmental pollution problem and protecting human health.

## 2. Material and Method

### 2.1. Preparation of the Biochar and Modified Biochar

Honeysuckle residue (HR) was obtained from a local pharmacy in Zhengzhou, China. The HR was dried at 105 °C for 2 h and smashed and passed through a 60 mesh sieve. The HR particles were calcinated in a tube furnace at 400 °C for 3 h in a N_2_ environment. The black matter was taken out, ground, and screened with a 100 mesh sieve in sequence.

Ferric chloride was added to a conical flask along with HDB. The optimum concentrations of Fe^3+^ and the solid–liquid ratio were determined to be 1 mol/L and 1:20 (W/V) in our pre-experiment, respectively. After vigorous stirring for 10 min, the mixture was oscillated in a shaker at 150 rpm for 8 h. The product was washed with deionized water until a constant pH was achieved. It was then dried at 60 °C for 12 h and sifted with a 100 mesh sieve to obtain the MHDB.

### 2.2. Characterization Techniques

The surface morphology of the HDB and MHDB before and after Cr(VI) adsorption was performed using a scanning electron microscope (SEM, Zeiss Genimi500, Zeiss, Barkhausenbau, Germany). The porosities and surface areas of the HDB and MHDB were measured using a surface area analyzer with a N_2_ atmosphere (BELSORP-max, MicrotracBEL, Osaka, Japan). The structures and crystallinities of the HDB and MHDB before and after Cr(VI) adsorption were characterized using an X-ray diffractometer (XRD, ZSX Primus II, Rigaku Corporation, Tokyo, Japan). The surface functional groups of HDB and MHDB before and after Cr(VI) adsorption were detected using Fourier transform infrared spectroscopy (FTIR, Thermo Fisher IS50, Thermo Fisher, Waltham, MA, USA). The elements and morphologies of the sample surface were analyzed using X-ray photoelectron spectroscopy (XPS, Thermo Scientific Escalab 250Xi+, Thermo Fisher, Waltham, MA, USA). The zeta potential of the MHDB was determined using a zeta potential meter after sonication (Particle Metrix GmbH, Particle Metrix, Munich, Germany).

### 2.3. Synthesis of Cr(VI)-Containing Wastewater

Cr(VI) solutions with different concentrations were synthesized by dissolving K_2_Cr_2_O_7_ into deionized water. The pH value of the wastewater was adjusted using 0.1 mol/L of NaOH or HCl. All chemicals used in the study were analytically pure.

### 2.4. Batch Adsorption Experiment

A total of 0.05 g biochar was added into 25 mL of Cr(VI) solution, and then the conical flasks were put into a temperature-controlled shaker and reacted at 120 rpm. At the end of the experiment, the mixture was filtered with a 0.45 μm filter membrane, the residual Cr(VI) was determined using diphenylcarbazide spectrophotometry at 540 nm. Each set was replicated three times, and the average value was used for subsequent analysis. The adsorption capacity was calculated by Equation (1), as follows:

(1) $$q=\frac{\left(C_{0}-C_{e}\right)V}{m}$$

where *q* (mg/g) is the adsorption capacity; *C*_0_ and *C_e_* (mg/L) are the initial and final concentration of Cr(VI), respectively; *m* (g) is the mass of biochar used for the adsorption; *V* (L) is the solution volume.

## 3. Results and Discussion

### 3.1. Sample Characterization

#### 3.1.1. Scanning Electron Microscope (SEM) Images

**Figure 1:**
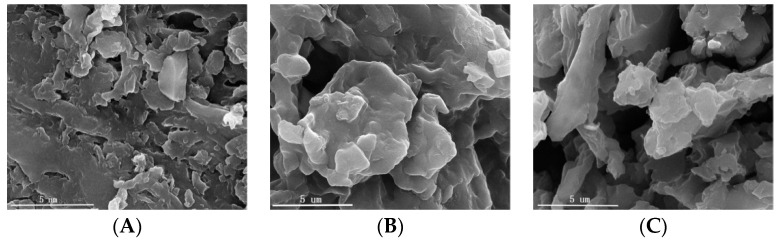
SEM images of (**A**) HDB, (**B**) MHDB, and (**C**) MHDB after adsorption of Cr(VI).

The SEM images of (A) HDB, (B) MHDB, and (C) MHDB after Cr(VI) adsorption are shown in Figure 1. The surface of the MHDB was rougher than that of the HDB due to the modification of ferric chloride (FeCl_3_); more surface pores in circular and irregular shapes appeared. Figure 1C shows that more irregular particles were dispersed on the surface after adsorption, which indicates that the Cr(VI) was successfully adsorbed onto the surface of the MHDB.

#### 3.1.2. Brunauer–Emmett–Teller (BET) Analysis

**Figure 2:**
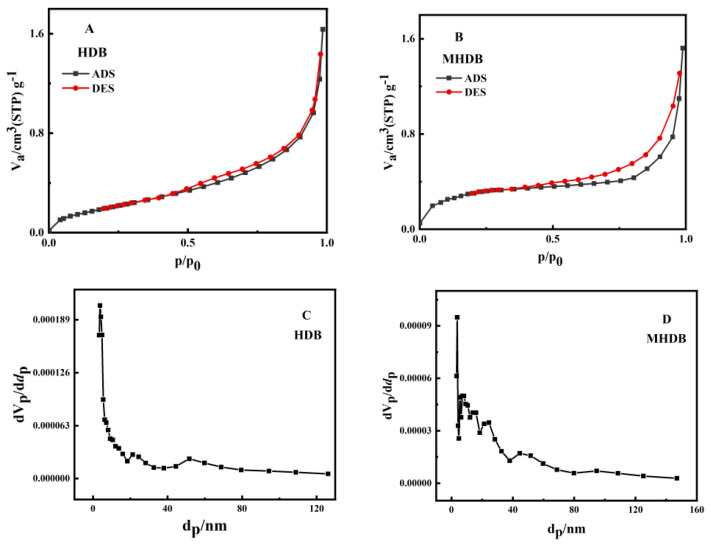
Nitrogen physisorption–desorption isotherms of HDB (**A**) and MHDB (**B**); BJH mesopore size distributions of HDB (**C**) and MHDB (**D**).

Figure 2 shows the N_2_ sorption–desorption isotherms of HDB and MHDB, which fitted the type IV isotherm, indicating the adsorbents are mesoporous materials. The average pore diameters of the HDB and MHDB were 12.45 nm and 8.33 nm, respectively. The hysteresis loop was at p/p_0_ > 0.4, as shown in Figure 2, indicating that the capillary condensation occurred in the biochar channels at relatively high pressures. The mesopore size distribution of the HDB was unimodal, with a peak pore size of 3.718 nm, as shown in Figure 2C. In addition, the peak pore size of the MHDB was 3.75 nm, and multiple peaks appeared, which indicates that the number of mesopores increased after modification. Therefore, the diffusion resistance decreased and the Cr(VI) diffused more easily into the pores of the adsorbent. The BET-specific surface areas of the HDB and MHDB were 0.81 m^2^/g and 1.13 m^2^/g, respectively, and the small specific surface area was mainly due to the raw material characteristics and the low sintering temperature. Considering the material properties, the mechanism of Cr(VI) adsorption might mainly be electrostatic adsorption, and the modification with iron enhanced the adsorption capacity of the adsorbent effectively.

#### 3.1.3. X-ray Diffractometer (XRD) Analysis

**Figure 3:**
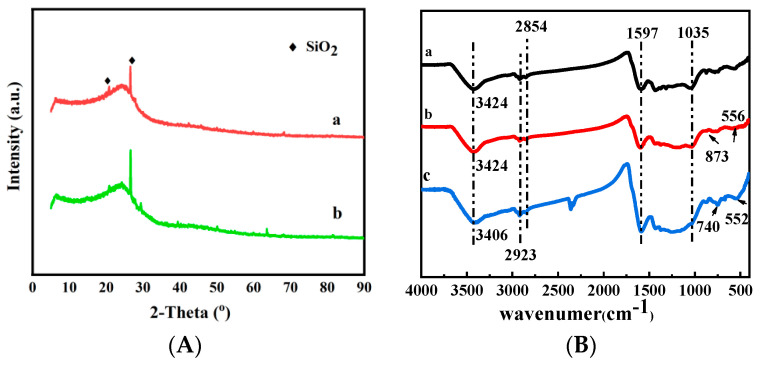
(**A**) The XRD patterns of (a) HDB and (b) MHDB; (**B**) FTIR spectra of (a) HDB, (b) MHDB; (c) MHDB after adsorption of Cr(VI).

For further study, the HDB and MHDB were characterized by XRD, and the results are shown in Figure 3A. The XRD patterns of the biochars show a large number of characteristic peaks, and the main peak is at 26.49°, indicating that the surface of the material contained crystalline material for SiO_2_. No obvious sharp peak appeared, indicating that the iron of the MHDB mainly existed with low crystallinity [40]. The small peaks of the MHDB at 2θ = 29.45°, 42.77°, and 60.05° indicate that the Fe_3_C particles may have been present in the MHDB, which was consistent with the XPS analysis results [41,42]. In addition, the modification of the ferric chloride (FeCl_3_) caused changes in the materials, according to Figure 1 and Figure 2. It is quite clear that the surface of the original HDB was smoother than that of the MHDB, and the average pore diameter increased after modification.

#### 3.1.4. Fourier Transform Infrared (FTIR) Analysis

Figure 3B shows that the wavenumber at 3424 cm^−1^ corresponds to the stretching oscillation of -OH, with a wide adsorption peak [43]. The wavenumbers at 2923 cm^−1^ and 2854 cm^−1^ could link to the symmetric and asymmetric stretching vibrations of C–H and C–C double bonds. The wavenumber at 1597 cm^−1^ is attributed to the stretching oscillation of C=C and C=O double bonds. The peak at 1035 cm^−1^ is assigned to the stretching vibrations of C–O. Except for the common functional groups, the oxygen-containing functional groups of the MHDB could have provided more chemical adsorption sites that improved the adsorption capacity, such as Ar-H, based on the wavenumber at 873 cm^−1^. Meanwhile, the wavenumber at 556 cm^−1^ is attributed to Fe–O, indicating that the adsorbent contained Fe [44]. Figure 3B shows that the peaks for the functional groups changed after Cr(VI) adsorption, as the peak around 3424 cm^−1^ (-OH ) shifted to 3406 cm^−1^, indicating that the functional groups were favorable for Cr(VI) removal. A new wavenumber at 2363 cm^–1^ appeared after adsorption, which corresponds to the formation of H_3_O^+^ [45]. In addition, the peak near 552 cm^−1^ (Fe–O) obviously increased after Cr(VI) adsorption. In summary, the change in the infrared spectrum after adsorption resulted from chemical denaturation, which indicates that the adsorption process was accompanied by a chemisorption reaction. The adsorption effect was closely related to the functional groups on the surface of the adsorbent, and the addition of functional groups by modification could have enhanced the adsorption.

#### 3.1.5. X-ray Photoelectron Spectroscopy (XPS) Analysis

**Figure 4:**
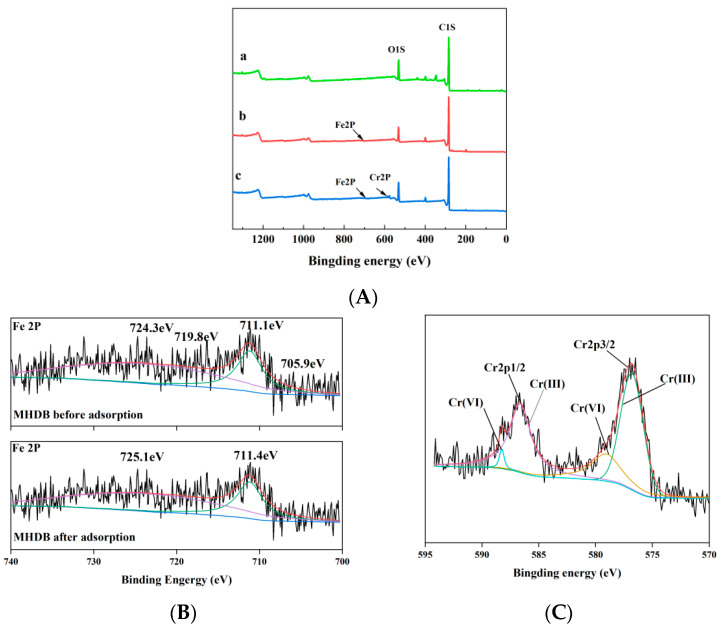
(**A**) The full XPS spectra of (a) HDB, (b) MHDB, (c) MHDB after adsorption; (**B**) detailed graph of the Fe 2p before and after adsorption; (**C**) detailed graph of the Cr 2p after adsorption.

The full XPS spectra of the (a) HDB, (b) MHDB, and (c) MHDB after adsorption of Cr(VI) are shown in Figure 4A. The C1s spectra of the HDB resolved into two peaks at 284 eV and 287.82 eV, representing the C–H and C=O, respectively [46,47]. The O1s spectra of the HDB resolved into two peaks at 532.29 eV and 533.1 eV, representing the -OH and C–O, respectively [48,49]. The binding energies and intensities of the peaks changed after modification, suggesting that the structure of the MHDB changed due to the iron modification. As shown in Figure 4B, the major peaks in the Fe 2p spectra are at 711.1 eV (Fe 2p3/2) and 724.3 eV (Fe 2p1/2) [50]. The distinctive satellite peaks at 705.9eV and 719.8eV are assigned to Fe_3_C, which is in agreement with a previous study [51]. After the adsorption of Cr(VI), the main peaks shifted to 711.4 eV and 725.1 eV, respectively, indicating that the iron was involved in the adsorption. As shown in Figure 4C, the binding energies at 576.8 eV (Cr 2p3/2) and 586.7 eV (Cr2p1/2) correspond to Cr(III), and the peaks of 579.0 eV (Cr 2p3/2) and 588.3 eV (Cr2p1/2) correspond to Cr(VI) [52,53]. Hence, the MHDB could have effectively reduced the Cr(VI) concentration in the water, converting the Cr(VI) to the stable Cr(III). At this point, the toxicity and harm of Cr(VI) to human health declines sharply after adsorption.

In sum, the sample characterization results suggest that the MHDB changed after the Cr(VI) adsorption, and the main mechanisms of the Cr(VI) adsorption might be physical adsorption (electrostatic interactions) and chemical adsorption (ion exchange, the reduction of Cr(VI) to Cr(III)).

### 3.2. Adsorption Study

#### 3.2.1. Effect of Initial Solution pH

**Figure 5:**
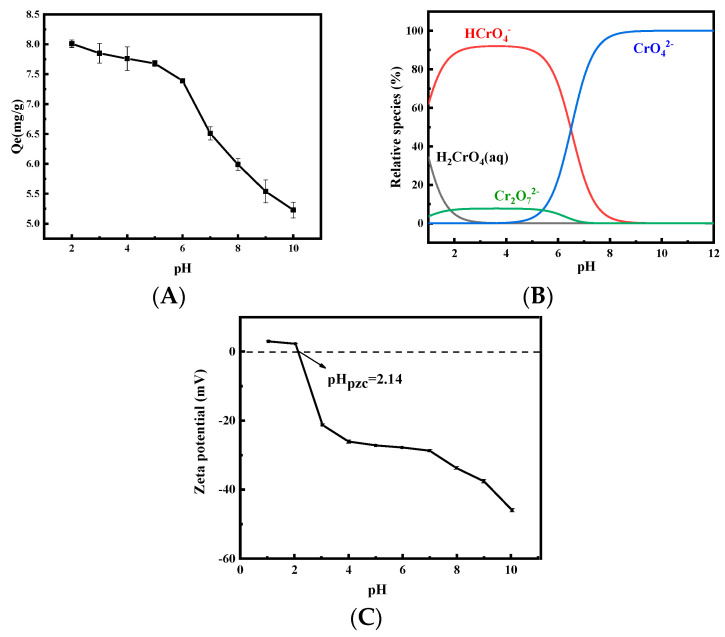
(**A**) Effect of initial solution pH on adsorption performance (*C*_0_ = 50 mg/L, time = 6 h, and temperature = 25 °C); (**B**) the existing forms of Cr(VI) in the solution at different pH levels; (**C**) zeta potential of MHDB.

The effect of the initial aqueous pH on the adsorption of Cr(VI) was studied by varying the initial pH from 2 to 10. Figure 5A showed the adsorption capacity of Cr(VI) by the MHDB decreased with the increasing pH, which is consistent with the results of a previous study [54]. The Cr(VI) adsorption capacity decreased slowly from 8.10 mg/g to 7.39 mg/g as the initial pH increased slowly from 2 to 6, and then the adsorption capacity decreased to 5.23 mg/g quickly as the initial pH rose to 10.

Previous studies have shown that the pH causes changes in the surface charge of the biochar and the degree of protonation of the functional groups on the biochar, affecting the form of Cr(VI) in the adsorption system significantly [55]. The main forms of chromium were CrO_4_^2−^ and HCrO_4_^−^ in the pH range of 2–8. Further research has shown that HCrO_4_^−^ was the main form, with pH values of 2–6.5, and the form of CrO_4_^2−^ mainly presented in water at pH values of >6.5 (Figure 5B) [56]. In addition, the free energy of the HCrO_4_^−^ was low, meaning it could be removed from the system more easily.

The dominant chemical reactions of the reduction of Cr(VI) to Cr(III) in the solution are expressed as Equations (2) and (3) [57]. As seen in Figure 5C, the pH_PZC_ value of the MHDB was measured to be 2.14, which indicates that the surface charge of the MHDB was positive at solution pH values lower than its pH_PZC_. The highest removal efficiency at a pH of 2 was due to the possible protonation of the adsorbent, which led to the weakening of the electrostatic repulsion and the enhancement of the electrostatic adsorption of Cr(VI) [58]. The protons in the solution were depleted, and the positive charge of the adsorbent surface turned into a negative charge as the initial pH increased; hence, the electrostatic attraction with Cr(VI) decreased. With the initial pH increase, the alkaline environment meant an increased presence of OH^−^, which indicates that the equilibrium in Equations (2) and (3) shifted to the left. That is, the alkaline environment suppressed the conversion reaction of Cr(VI) to Cr(III) and was detrimental to the removal of Cr(VI). The experiment results indicate that a lower pH was beneficial for Cr(VI) removal, and a pH of 2 was best for Cr(VI) adsorption by MHDB in the pH range of 2–10. Therefore, the severe threat of Cr(VI) to human health is mitigated by MHDB more effectively in an acidic environment.

Cr_2_O_7_^2−^ + 14H^+^ + 6e^−^ → 2Cr^3+^ + 7H_2_O (2)

HCrO_4_^−^ + 7H^+^ + 3e^−^ → Cr^3+^ + 4H_2_O (3)

#### 3.2.2. Effect of Adsorption Time and Adsorption Kinetics

**Figure 6:**
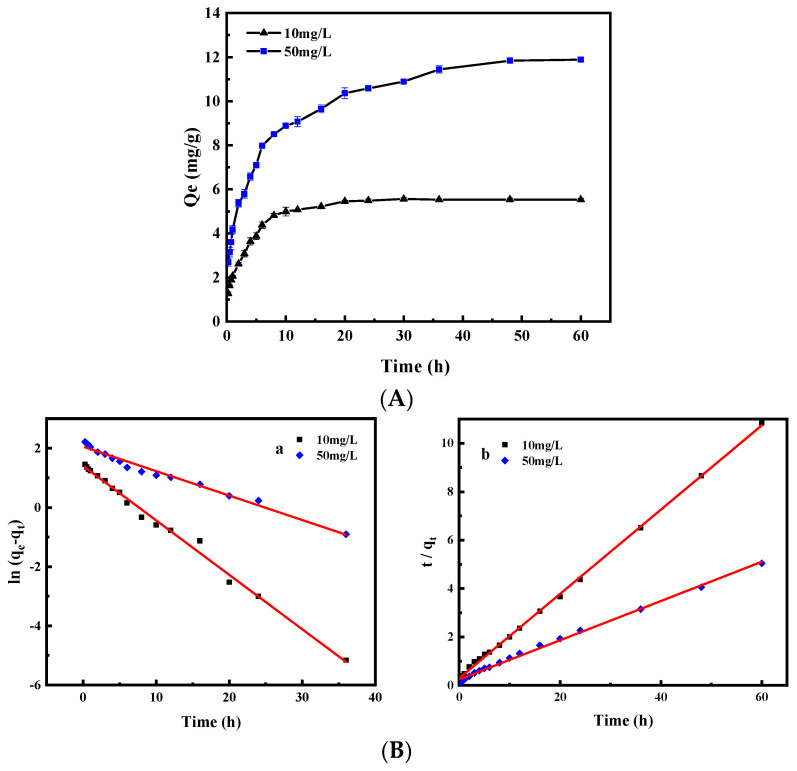
(**A**) Effect of adsorption time on adsorption performance (*C*_0_ = 10 and 50 mg/L, pH = 2, and temperature = 25 °C); (**B**) Linear kinetic plots of pseudo-first model (**a**) and pseudo-second model (**b**) for Cr(VI) adsorption by MHDB.

The adsorption of Cr(VI) was affected by the adsorption time, and the result is illustrated in Figure 6A. It can be seen that Cr(VI) adsorption increased with the increase in reaction time, and the Cr(VI) adsorption took place in two stages. The adsorption capacities in the first 12 h rapidly reached 91.65% and 76.65% of the equilibrium adsorption capacity with the initial Cr(VI) concentrations of 10 and 50 mg/L, respectively. In the second stage, the removal rate showed slow growth, and the adsorption of Cr(VI) reached equilibrium at 48 h. The active sites of the adsorbent were unbound in the early stage, meaning it could combine with Cr(VI) quickly. Most of the active sites were occupied along with the reaction time, which led to slow adsorption and the equilibrium of the adsorption at 48 h.

Pseudo-first-order and pseudo-second-order models were used to fit the data obtained from the experiment [59]. The two Equations (4) and (5) are expressed as follows:

(4) $$ln\left(q_{e}-q_{t}\right)=lnq_{e}-k_{1}t$$

(5) $$\frac{t}{q_{t}}=\frac{t}{q_{e}}+\frac{1}{k_{2}q_{e}^{2}}$$

where *q_t_* and *q_e_* (mg/g) are the Cr(VI) uptakes at time *t* and at equilibrium time, respectively; *k*_1_ (h^−1^) is the equilibrium rate constant, and *k*_2_ [g/(mg·h)] is the equilibrium rate constant, respectively.

Figure 6B shows the linear kinetic plots of the two models, and the kinetics parameters for the adsorption are listed in Table 1. It can be seen that the regression coefficient of the pseudo-second-order equation is higher out of the two equations (R^2^ > 0.993). Moreover, the adsorption amounts calculated based on the pseudo-second-order equation at a different initial concentration of Cr(VI) were 5.74 and 12.33 mg/g, which are in good agreement with the experimental data at 5.54 and 11.84 mg/g, respectively. In sum, the pseudo-second-order equation was better to describe the adsorption process of Cr(VI) by MHDB, and the human health impact of Cr(VI) was weakened after adsorption.

#### 3.2.3. Adsorption Isotherms

**Figure 7:**
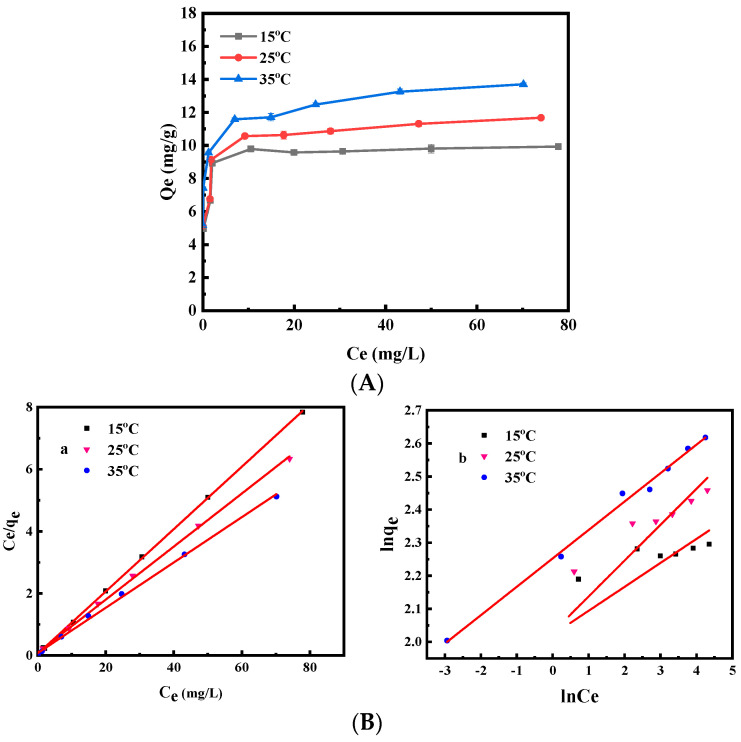
(**A**) Isotherms of Cr(VI) adsorption removal by MHDB (*C*_0_ = 10–100 mg/L, pH = 2, time = 48 h, and temperature = 15, 25 and 35 °C); (**B**) adsorption isotherms of Cr(VI) by fitting the Langmuir isotherm model (**a**) and Freundlich isotherm model (**b**).

As the initial Cr(VI) concentration increased from 10 to 100 mg/L, the uptake of Cr(VI) by MHDB increased from 4.96 to 9.93 mg/g, from 5.13 to 11.68 mg/g, and from 5.17 to 13.70 mg/g at 15, 25, and 35 °C, respectively (Figure 7A). The results might be attributed to the fact that the concentration difference between the adsorbent and the solution obviously increased when the initial Cr(VI) concentration increased. In this respect, the difference accelerated the mass transfer rate and promoted the adsorption reaction of Cr(VI).

Figure 7A shows the relationship between the Cr(VI) concentration and the adsorption capacity of the MHDB at equilibrium. In addition, the adsorption isotherm data were fitted by the Langmuir and Freundlich models [60]. The linearized forms of the two models are expressed by Equations (6) and (7) below:

(6) $$\frac{C_{e}}{q_{e}}=\frac{1}{Q_{m}K_{L}}+\frac{C_{e}}{Q_{m}}$$

(7) $$lnq_{e}=lnK_{f}+\frac{1}{n}lnC_{e}$$

where *C_e_* (mg/L) and *q_e_* (mg/g) are the concentration and adsorption capacity at equilibrium, respectively; *K_L_* (L/mg) is the Langmuir constant related to the binding energy, and *Q_m_* (mg/g) is the maximum adsorption capacity; *K_f_* is the Freundlich constant related to the adsorption capacity, and *n* is the adsorption intensity.

Figure 7B shows the fitting results of the Langmuir and Freundlich models, and the isotherm parameters are listed in Table 2. The regression coefficients R^2^ of the Langmuir equation were higher than those of the Freundlich equation at temperatures of 15, 25, and 35 °C, indicating that the Langmuir equation was more suitable for describing the adsorption process (R^2^ > 0.997), and the adsorption of Cr(VI) by MHDB had a monolayer adsorption nature. Though other isotherm models might offer further insight, considering that the Langmuir equation was significantly suitable for the adsorption process, other isotherm models were no longer used for analysis in this study. Based on the Langmuir isotherm model, the calculated maximum adsorptions were 9.95, 11.70, and 13.70 mg/g at 15, 25, and 35 °C, respectively. In sum, the impact of Cr(VI) on human health declined more easily by MHDB at higher temperatures.

The maximum adsorption capacity of the MHDB was about 13.70 mg/g at 35 °C, which was higher than those of multiple other types of biochars from previous studies. For instance, the adsorption capacities of Cr(VI) by the pineapple peel-derived biochar, the natural zeolite-derived biochar, the nano-magnetite-modified biochar material, and the cornstalk-derived biochar were 7.44 mg/g, 6.08 mg/g, 9.92 mg/g, and 9.25 mg/g, respectively [61,62,63,64]. Thus, the modified honeysuckle-derived biochar is a promising adsorbent for Cr(VI) removal and could be a valuable reference for the resource recovery of TCMRs, alleviating the environmental pollution problem from the chromium-containing wastewater.

#### 3.2.4. Adsorption Thermodynamics

The thermodynamics of Cr(VI) adsorbed by MHDB were studied at 288 K, 298 K, and 308 K, and the parameters were calculated using Equations (8)–(10), as follows [65]:

(8) $$\Delta G^{0}=\Delta H^{0}-T\Delta S^{0}$$

(9) $$lnK_{d}=\frac{\Delta S^{0}}{R}+\frac{\Delta H^{0}}{RT}$$

(10) $$K_{d}=\frac{q_{e}}{C_{e}}$$

where *K_d_* is the distribution coefficient (L/g); the *K_d_* value is obtained by plotting ln(*q_e_*/*C_e_*) against *C_e_* and extrapolating to zero *C_e_*; Δ*S*^0^ is the standard entropy change (kJ/mol·K); Δ*H*^0^ is the standard enthalpy change (kJ/mol); *R* is the ideal gas constant (8.314J mol/k); *T* is the reaction temperature (*K*); Δ*G*^0^ is the standard Gibbs free energy change (kJ/mol); *C_e_* is the concentration of Cr(VI) at equilibrium (mg/L); *q_e_* is the adsorption capacity of MHDB on Cr(VI) at equilibrium (mg/g).

**Figure 8:**
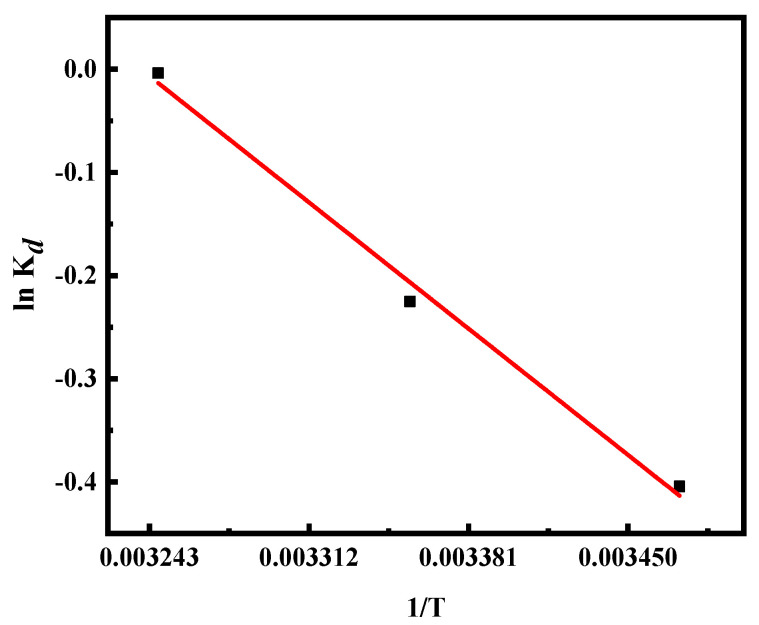
Thermodynamics plot for adsorption of Cr(VI) by MHDB.

Figure 8 shows the relationship between the 1/*T* and ln*K_d_*. The values of Δ*S*^0^, Δ*H*^0^, and Δ*G*^0^ are shown in Table 3. It can be seen that the adsorption process had a spontaneous character since all three values of Δ*G*^0^ at 288, 298, and 308 *K* were negative. Meanwhile, the spontaneous nature of the adsorption process increased since the value of Δ*G*^0^ decreased gradually as the temperature increased. The higher temperature was favorable for the adsorption of Cr(VI) by MHDB due to the fact that the high reaction temperature decreased the solution viscosity and the diffusion resistance of Cr(VI) ions, which could speed up the adsorption process. Thus, the higher temperature was beneficial for lessening the harmful effects of Cr(VI) on human health.

#### 3.2.5. Effect of Co-Existing Ions

**Figure 9:**
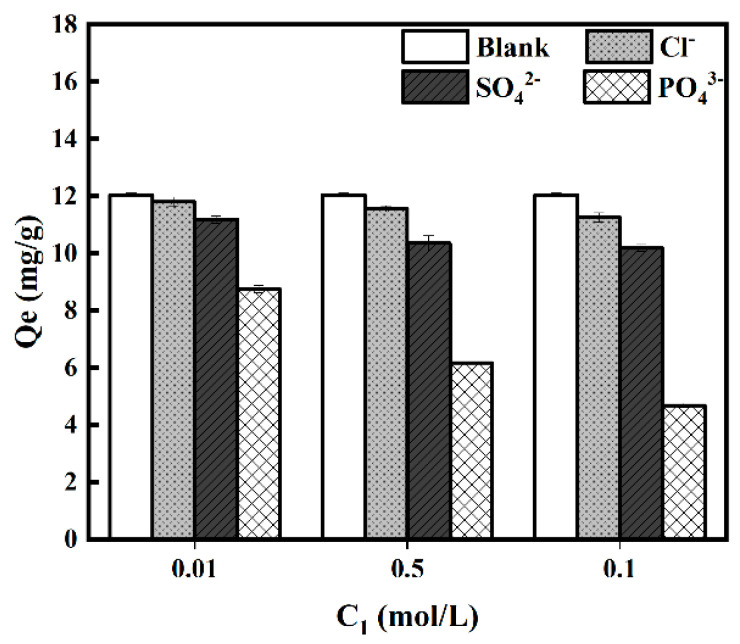
Effect of co-existing ions on adsorption performance (*C*_0_ = 150 mg/L, time = 6 h, pH = 2, temperature= 25 °C).

In general, wastewater may contain other anions that could influence the adsorption of Cr (VI). Therefore, the impact of co-existing ions on the adsorption of Cr(VI) was investigated in this study, and the results are shown in Figure 9. It can be seen that the adsorption capacity of Cr(VI) decreased with the presence of anions, and the effect of PO_4_^3^^−^ was the most significant among the three anions. In addition, the adsorption capacity of Cr(VI) decreased with the increase of the anion concentration. As shown in Figure 9, the PO_4_^3^^−^ with a high concentration (0.1 mol/L) significantly inhibited Cr(VI) removal, and the adsorption capacity of Cr(VI) decreased to 4.68 mg/g—a decrease of 61.20%.

The competition between anions and Cr(VI) for adsorption might mainly be due to their similar ionic nature; the anions occupied the active sites on the surface of the adsorbent and formed complexes [66,67]. The interference was directly proportional to the charge on the anions; the anionic charges provided by the anions Cl^−^, SO_4_^2^^−^, and PO_4_^3^^−^ were −1, −2, and −3, respectively, and hence, the negative effects on the Cr(VI) removal efficiency followed the order of Cl^−^ < SO_4_^2^^−^ < PO_4_^3^^−^. Overall, these results suggest that the presence of anions could affect the removal of Cr(VI) by MHDB, and the impact was positively related to the charges and concentrations of the anions. Therefore, the anions reduced the adsorption capacity of the MHDB for Cr(VI); in other words, they increased the harmful effects of Cr(VI) on human health.

## 4. Conclusions

Hexavalent chromium (Cr(VI)) is a severe threat to human health. In order to solve the hazard of chromium in the environment, the removal of Cr(VI) using Fe^3+^-modified honeysuckle-derived biochar (MHDB) was researched in this study. The highest Cr(VI) removal was observed at a pH of 2 in the initial pH range of 2–10. The adsorption of Cr(VI) took place in two stages; the removal efficiency increased rapidly in the first 12 h, and then increased slowly until the equilibrium was reached. The adsorption process followed pseudo-second-order kinetics and the Langmuir isotherm. The thermodynamic study showed that the adsorption process was spontaneous and endothermic. The anions had negative effects on Cr(VI) adsorption in the order of Cl^−^ < SO_4_^2^^−^ < PO_4_^3^^−^. Based on the experimental results, the MHDB reduced the Cr(VI) concentration in water distinctly, demonstrating a high adsorption capacity and an efficient ability to convert the Cr(VI) to the Cr(III). In general, this study provides a new material for the adsorption of Cr(VI) and has important reference value for mitigating the harm of chromium pollution and protecting environmental health.

## Figures and Tables

**Table 1:** The kinetics parameters for the adsorption of Cr(VI) by MHDB.

*C*_0_ mg/L	Pseudo-First-Order			Pseudo-Second-Order		
	*q_e_*	*k*_1_	*R* ^2^	*q_e_*	*k*_2_	*R* ^2^
10	4.05	0.1838	0.9925	5.74	0.1741	0.9991
50	7.78	0.0826	0.9789	12.33	0.0811	0.9962

**Table 2:** The parameters of Langmuir equation and Freundlich equation.

Reaction Temperature °C	Langmuir Equation			Freundlich Equation		
	*Q_m_*	*K_L_*	*R* ^2^	*K_f_*	1/*n*	*R* ^2^
15	9.95	2.039	0.9998	7.551	0.0724	0.5089
25	11.70	0.972	0.9989	7.605	0.1085	0.7089
35	13.70	0.979	0.9979	9.511	0.0859	0.9924

**Table 3:** Thermodynamic parameters for adsorption of Cr(VI) by MHDB.

Reaction Temperature	Δ*G*^0^	Δ*H*^0^	Δ*S*^0^
288 K	−0.15	14.74	51.73
298 K	−0.67		
308 K	−1.19		
